# Supplementary material for: Comparison of cryptobenthic reef fish communities among microhabitats in the Red Sea
Source: PeerJ. 2018 Jun 18;6:e5014. doi: 10.7717/peerj.5014 (PMC6011822; doi:10.7717/peerj.5014)
Supplement: Supplemental Information 1 — List of identification keys and guides used to identify fishes collected from the central Saudi Arabian Red Sea. [file peerj-06-5014-s001.docx]

Delventhal NR, Mooi RD, Bogorodsky SV, Mal AO (2016) A review of the *Callogobius* (Teleostei: Gobiidae) from the Red Sea with the description of a new species. Zootaxa 4179:225-243

Greenfield DW (2016) A key to the dwarfgoby species (Teleoseti: Gobiidae: *Eviota*) described between 1871 and 2016. J Ocean Sci Found 24:35-90

Herler J, Hilgers H (2005) A synopsis of coral and coral-rock associated gobies (Pisces: Gobiidae) in the Gulf of Aqaba, northern Red Sea. Aqua 10:103-132

Larson HK, Murdy EO (2001) Gobiidae. Gobies. Pp 3578-3603. In: Carpenter KE, Niem VH (eds) FAO species identification guide for fishery purposes. The living marine resources of the western Central Pacific. Volume 6. Bony fishes part 4 (Labridae to Latimeriidae). FAO, Rome

Murdy EO, Hoese DF (1985) Revision of the gobiid fish genus *Istigobius*. Indo-Pac Fishes 4:1-41

Randall JE, Greenfield DW (2001) A preliminary review of the Indo-Pacific gobiid fishes of the genus *Gnatholepis.* Ichthyol 69:1-17
