# Supplementary material for: Comparison of cryptobenthic reef fish communities among microhabitats in the Red Sea
Source: PeerJ. 2018 Jun 18;6:e5014. doi: 10.7717/peerj.5014 (PMC6011822; doi:10.7717/peerj.5014)
Supplement: Supplemental Information 2 — Percent cover for each quadrat is separated into the habitat components found within as seen from an aerial view. A rugosity value of 1 indicates a completely flat surface while higher values represent greater vertical complexity. [file peerj-06-5014-s002.docx]

| **Habitat** | **Quadrat** | **Percent Cover** | | | | | **Rugosity** | **Depth (m)** |
| --- | --- | --- | --- | --- | --- | --- | --- | --- |
|  |  | ***Acropora* spp.** | **Other coral** | **Live rock** | **Rubble** | **Sand** |  |  |
| **Coral** | 1  2  3  4  5 | 23  49.5  63.5  64  82.3 | 64.5  40.7  14.9  11  12.9 | 12.5  7.8  -  -  - | -  -  7.6  5  2.8 | -  2  14  20  2 | 1.26  1.19  1.32  1.23  1.42 | 10  12  12  13  12 |
| **Rubble** | 1  2  3  4  5 | -  -  -  -  - | -  -  -  -  - | -  -  -  -  - | 62.5  62.6  53.1  70.3  79.6 | 37.5  37.4  46.9  29.7  20.4 | 1.07  1.11  1.07  1.10  1.11 | 13  13  12  12  12 |
| **Sand** | 1  2  3  4  5 | -  -  -  -  - | -  -  -  -  - | -  -  -  -  - | 4.9  1  1.8  2  2 | 95.1  99  98.2  98  98 | 1.02  1.03  1.01  1.00  1.01 | 13  12  15  12  12 |
