# Supplementary material for: Comparison of cryptobenthic reef fish communities among microhabitats in the Red Sea
Source: PeerJ. 2018 Jun 18;6:e5014. doi: 10.7717/peerj.5014 (PMC6011822; doi:10.7717/peerj.5014)
Supplement: Supplemental Information 3 — Species list and counts of all 326 fishes collected in the central Saudi Arabian Red Sea using 1m2 rotenone stations in three microhabitat types. Asterisk denotes Red Sea endemic species. [file peerj-06-5014-s003.docx]

| **Family** | **Species** | **Coral** | **Rubble** | **Sand** | **Total** |
| --- | --- | --- | --- | --- | --- |
| Apogonidae | *Apogon* sp. 1 | 1 | - | - | 1 |
|  | *Cheilodipterus pygmaios** | 1 | 3 | - | 4 |
|  | *Fowleria* sp. 1 | 1 | - | 1 | 2 |
| Blenniidae | *Ecsenius aroni** | 1 | 1 | - | 2 |
|  | *Ecsenius frontalis** | - | 1 | - | 1 |
| Bothidae | Bothidae sp. 1 | - | - | 1 | 1 |
| Callionymidae | Callionymidae sp. 1 | - | - | 5 | 5 |
| Chaetodontidae | *Chaetodon austriacus** | 1 | - | - | 1 |
| Gobiidae | *Amblygobius albimaculatus* | - | - | 1 | 1 |
|  | *Asterropteryx semipunctata* | 1 | 26 | - | 27 |
|  | *Bryaninops natans* | 1 | - | - | 1 |
|  | *Callogobius amikami** | 1 | 1 | - | 2 |
|  | *Callogobius bifasciatus* | 2 | 26 | 1 | 29 |
|  | *Eviota distigma* | 6 | 8 | 3 | 17 |
|  | *Eviota guttata* | - | 1 | - | 1 |
|  | *Eviota punyit* | - | 1 | - | 1 |
|  | *Eviota* sp. 1 | - | 5 | - | 5 |
|  | *Eviota* sp. 2 | - | 1 | - | 1 |
|  | *Eviota* sp. “Red Sea 3” | 1 | 6 | 1 | 8 |
|  | *Eviota zebrina* | 7 | 15 | - | 22 |
|  | *Gnatholepis anjerensis* | - | 2 | - | 2 |
|  | Gobiidae sp. 1 | - | 1 | - | 1 |
|  | Gobiidae sp. 2 | - | 9 | 2 | 11 |
|  | Gobiidae sp. 3 | - | 1 | - | 1 |
|  | Gobiidae sp. 4 | - | 3 | - | 3 |
|  | Gobiidae sp. 5 | - | 1 | 1 | 2 |
|  | Gobiidae sp. 6 | - | 1 | 1 | 2 |
|  | Gobiidae sp. 7 | - | 2 | - | 2 |
|  | Gobiidae sp. 8 | - | 1 | - | 1 |
|  | Gobiidae sp. 9 | - | 1 | - | 1 |
|  | *Gobiodon citrinus* | - | - | 1 | 1 |
|  | *Gobiodon reticulatus** | 2 | 1 | - | 3 |
|  | *Gobiodon rivulatus* | - | - | 1 | 1 |
|  | *Istigobius decoratus* | 1 | 10 | 9 | 20 |
|  | *Koumansetta hectori* | 4 | 4 | 1 | 9 |
|  | *Lotilia graciliosa* | - | 3 | - | 3 |
|  | *Pleurosicya prognatha* | 3 | - | 1 | 4 |
|  | *Trimma avidori** | 17 | 26 | 1 | 44 |
|  | *Trimma flavicaudatum** | 1 | 5 | - | 6 |
| Labridae | *Hemigymnus fasciatus* | - | 1 | - | 1 |
|  | *Larabicus quadrilineatus** | 1 | - | - | 1 |
|  | *Pseudocheilinus hexataenia* | 3 | 1 | 1 | 5 |
|  | *Pteragogus cryptus* | 1 | - | - | 1 |
|  | *Wetmorella nigropinnata* | 1 | - | - | 1 |
| Opistognathidae | Opistognathidae sp. 1 | - | 1 | - | 1 |
|  | Opistognathidae sp. 2 | - | 6 | 1 | 7 |
| Pinguipedidae | *Parapercis hexophtalma* | - | 1 | - | 1 |
| Pomacanthidae | *Centropyge multispinis* | 1 | - | - | 1 |
| Pomacentridae | *Amblyglyphidodon flavilatus** | 5 | 4 | - | 9 |
|  | *Chromis viridis* | - | 1 | - | 1 |
|  | *Dascyllus aruanus* | - | 1 | - | 1 |
|  | [*Plectroglyphidodon lacrymatus*](http://www.fishbase.org/summary/SpeciesSummary.php?id=5712) | - | 1 | - | 1 |
|  | Pomacentridae sp. 1 | 1 | - | - | 1 |
|  | Pomacentridae sp. 2 | 2 | - | - | 2 |
|  | Pomacentridae sp. 3 | - | 1 | - | 1 |
| Pseudochromidae | *Chlidichthys auratus** | - | 2 | - | 2 |
|  | *Chlidichthys* cf. *rubiceps** | 1 | 1 | - | 2 |
|  | Congrogadinae sp. 1 | - | 1 | - | 1 |
|  | Pseudochromidae sp. 1 | - | 1 | - | 1 |
|  | Pseudochromidae sp. 2 | 11 | - | - | 11 |
|  | *Pseudochromis flavivertex** | - | 7 | - | 7 |
|  | *Pseudochromis olivaceus** | - | - | 1 | 1 |
|  | *Pseudochromis* sp. 1 | - | 2 | - | 2 |
|  | *Pseudochromis* sp. 2 | - | 3 | - | 3 |
|  | *Pseudochromis* sp. 3 | 1 | - | - | 1 |
|  | *Pseudochromis* sp. 4 | - | - | 1 | 1 |
|  | *Pseudochromis springeri** | 1 | - | - | 1 |
| Syngnathidae | Syngnathinae sp. 1 | - | - | 1 | 1 |
|  | Syngnathinae sp. 2 | - | 1 | - | 1 |
| Synodontidae | *Saurida gracilis* | - | 1 | - | 1 |
| Tetraodontidae | *Canthigaster pygmaea** | 1 | 1 | - | 2 |
| Tripterygiidae | Tripterygiidae sp. 1 | - | 3 | - | 3 |
| Xenisthmidae | *Xenisthmus* cf. *balius** | - | 1 | - | 1 |
