# Supplementary figures and images for: Comparison of cryptobenthic reef fish communities among microhabitats in the Red Sea
Source: PeerJ. 2018 Jun 18;6:e5014. doi: 10.7717/peerj.5014 (PMC6011822; doi:10.7717/peerj.5014)

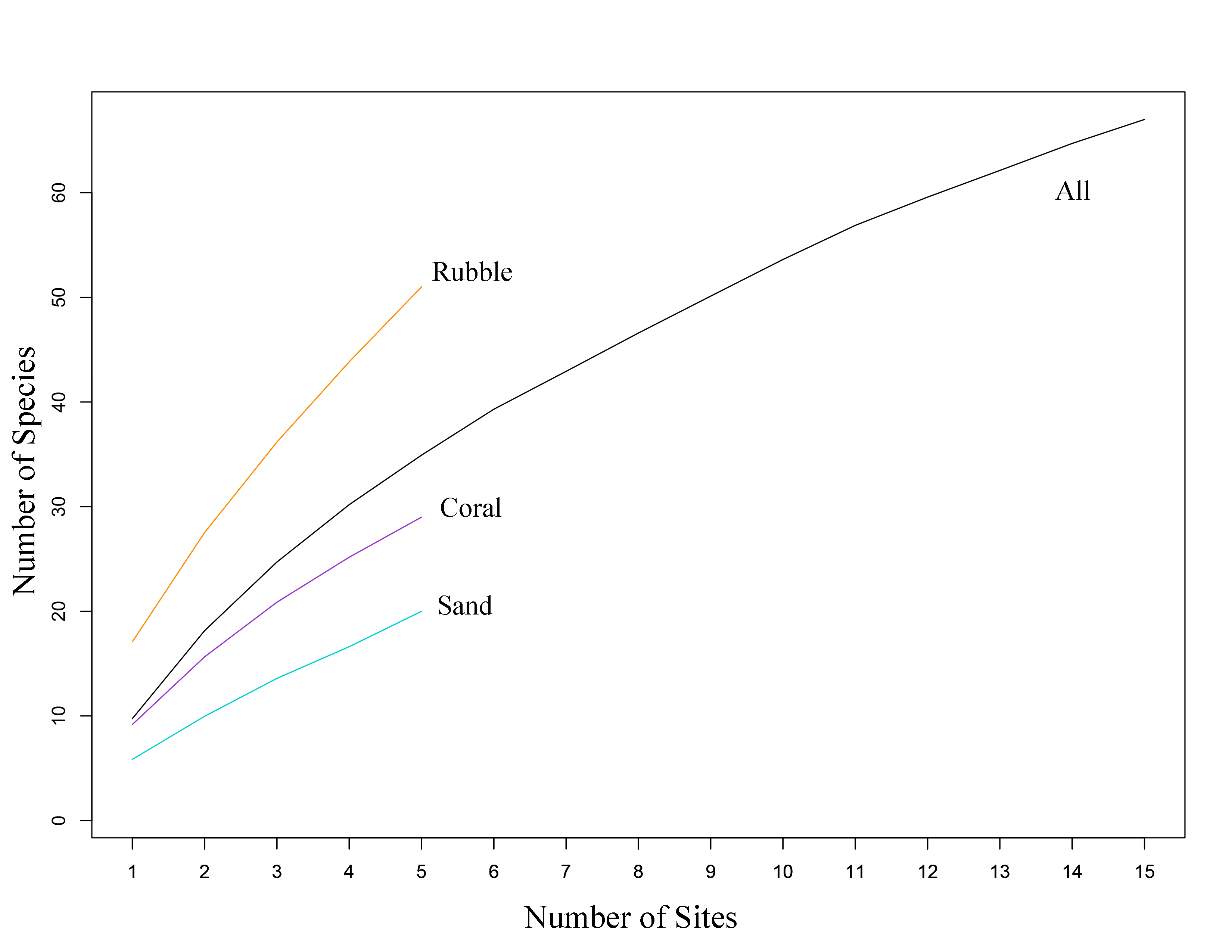

Supplement: Supplemental Information 4 — Fishes were sampled from three microhabitat types (n = 5 quadrats per microhabitat type) in the central Saudi Arabian Red Sea. 71 species of fish were recorded from 15 1 m2 quadrats. [file peerj-06-5014-s004.png]
